# Sini-san improves duodenal tight junction integrity in a rat model of functional dyspepsia

**DOI:** 10.1186/s12906-017-1938-2

**Published:** 2017-08-30

**Authors:** Xiongfei Chang, Luqing Zhao, Jiajia Wang, Xiaofang Lu, Shengsheng Zhang

**Affiliations:** 1 Beijing University of Chinese Medicine, Beijing, China; 2 Digestive Disease Center, Beijing Hospital of Traditional Chinese Medicine Affiliated to Capital Medical University, Beijing, China

**Keywords:** Sini-san, Functional dyspepsia, Duodenum, Tight junction, Pro-inflammatory cytokine

## Abstract

**Background:**

Recent reports have demonstrated that impaired barrier function and local microinflammation in the duodenal mucosa contribute to the pathogeneses of functional dyspepsia (FD). Thus, restoring normal barrier integrity becomes a potential therapeutic strategy in the treatment of FD. Sini-San (SNS) is a traditional Chinese prescription that exhibits therapeutic effects in FD, but the underlying mechanisms remain not well understood.

**Methods:**

FD rats were established by tail clamping method and the therapeutic effect of SNS was evaluated by measuring the visceral sensitivity and gastric compliance. Transepithelial electrical resistance (TEER) that reveals epithelial barrier integrity was measured by Ussing chamber. The expression of tight junction (TJ) proteins, occludin and claudin-1, in the duodenum was determined by Western blot and immunofluorescence. The amount of tumor necrosis factor alpha (TNF-α) and interferon gamma (INF-γ) in duodenal mucosa was detected by enzyme-linked immune sorbent assay (ELISA). The mRNA level of transient receptor potential vanilloid type 1 (TRPV1) was measured by quantitative real time-polymerase chain reaction (qPCR).

**Results:**

SNS could improve gastric compliance and attenuate visceral hypersensitivity (VH) in FD rats. TEER was decreased in FD rats, but treatment with SNS restored normal level of TEER and the expression of occludin and claudin-1 in FD rats. In addition, SNS administration ameliorated FD-associated increase in the production of TNF-α, IFN-γ and the expression of TRPV1.

**Conclusions:**

The therapeutic effect of SNS on FD is at least partially through improvement of TJ integrity and attenuation of FD-associated low-grade inflammation in the duodenum. Our findings highlight the molecular basis of SNS-based treatment of FD in human patients.

**Electronic supplementary material:**

The online version of this article (10.1186/s12906-017-1938-2) contains supplementary material, which is available to authorized users.

## Background

Functional dyspepsia (FD) is a common functional gastrointestinal disorder affecting 10–30% of the population globally [1]. FD is characterized by upper abdominal pain, early satiety and belching. The underlying pathogenesis is complicated and largely unknown. Potential causes are visceral hypersensitivity (VH), decreased gastric compliance and abnormal gastric motility. It was proposed there are no structural changes associated with FD in the upper gut, but recent studies have shown that duodenal barrier function is impaired and low-grade inflammation is present in the duodenum of FD patients. These findings recognize that the duodenum has a key role in the pathogenesis of FD [2, 3], and propose that duodenal barrier dysfunction and local low-grade inflammation are potential pathogenic factors for FD.

The intestinal barrier plays a critical role in preventing the translocation of noxious substances from the gut lumen to the submucosa, and the tight junction (TJ) proteins are key structural factors of this barrier. Dysfunction in intestinal barrier causes abnormal penetration of toxic substances, which results in increased local expression of inflammatory factors or infiltration of inflammatory cells. Many studies have proved that this pathological process often occurs in inflammatory bowel disease [4].

Sini-San (SNS) is a traditional Chinese prescription that has been widely applied in the treatment of various gastrointestinal diseases, including FD [5–7]. SNS is composed of four herbs, Chaihu (*Radix Bupleuri Chinensis*), Baishao (*Radix Paeoniae Alba*), Zhishi (*Fructus Aurantii Immaturus*), and Gancao (*Radix Glycyrrhizae*). It was shown that SNS could reduce the permeability of intestinal mucosa [8], and pharmacological studies also demonstrated that some extracts of these herbs, such as 18β-glycyrrhetinic acid [9], could also protect or repair intestinal epithelial TJ. However, it is unknown whether SNS plays a therapeutic role in FD by repairing tight junction integrity in the duodenum.

The aims of this study were to evaluate the therapeutic effect of SNS in FD and to determine whether SNS regulates the expression of TJ proteins thereby improving duodenal barrier function.

## Methods

### SNS preparation

SNS consists of Chaihu (*Radix Bupleuri Chinensis,* derived from *Bupleurum chinense DC, voucher number 16012002*), Baishao (*Radix Paeoniae Alba,* derived from *Paeonia lactiflora Pall, voucher number 16040201*), Zhishi (*Fructus Aurantii Immaturus,* derived from *Citrus aurantium L, voucher number 16011370*), and Gancao (*Radix Glycyrrhizae,* derived from *Glycyrrhiza uralensis Fisch, voucher number 15091001*) with a ratio of 1:1:1:1.

These raw herbs were purchased from Beijing Xinglin Pharmaceutical Company and were identified as eligible medicinal material. SNS was prepared by the Beijing Hospital of Traditional Chinese Medicine Affiliated to Capital Medical University. Specifically, a total weight of 400 g of the above raw herbs was mixed and impregnated in 2400 ml distilled water for 30 min. Then the medical materials were boiled for 30 min and 400 ml SNS preparation was harvested. This procedure was repeated, and a final volume of 800 ml was obtained from each 400 g herbs. The decoction was stored at 4 °C until the experiment. The major components of SNS decoction were saikosaponins, peoniflorin, naringin and glycyrrhizic acid [10–12].

### Animals

Thirty six healthy male Sprague-Dawley rats (SPF grade) weighing 200 ± 20 g were bought from Beijing Vital River Laboratory Animal Technology Company. The rats were housed in cages maintained under a 12-h light/12-h dark cycle with the room temperature of 22 ± 1 °C and a humidity of 65–70%. All of the rats had free access to food and water. Twenty four rats were used to establish the FD model by tail clamping approach as previously described [13], and the remaining 12 rats were divided into control group. In brief, every four rats were kept in a cage, and a surgical forcep was used to clamp the distal one third of the tail. This tail clamping was practiced every 4 h with a duration of 30 min, and 3 times per day for 7 days. The FD rats were then randomly divided into two groups that receive SNS or water as a vehicle control. Equal volume of SNS or water was given through gavage at the dose of 1 ml/100 g body weight for 7 consecutive days. At the end of treatment, 6 rats of each group were used to evaluate the gastric compliance and sensitivity. The remaining 6 rats in each group were anesthetized using 2% pentobarbital sodium and the duodenum was excised for the following experiments.

Animal experiments were performed in accordance with the Guide for the Care and Use of Laboratory Animals published by the National Institutes of Health (NIH Publications No. 85–23, revised 1996) and approved by the Animal Care and Use Committee of China Academy of Traditional Chinese Medicine.

### Gastric distension procedure and myoelectricity record

Gastric compliance and sensitivity were evaluated by using barostat and electrophysiological recorder as reported previously [14, 15]. Briefly, the rats were temporarily anesthetized by isoflurane. A polyethylene balloon (maximum volume 20 ml) with a polyvinyl tube was introduced from the mouth to the stomach, and two electrodes were fixed into the trapezius of the rat. The balloon and the electrodes were connected to the barostat and electrophysiological recorder respectively. The experiments began when the rats were conscious. The pressure of the balloon was increased stepwise, 20, 40, 60, 80 mmHg, with duration of 30 s and at an interval of 3 min between distensions. The volume of the balloon was recorded by the Protocol PlusDeluxe 9.6 R (G&J Electronics, Canada). Simultaneously, the myoelectricity was recorded by the LabChart (AD instrument, Australia). The gastric compliance was calculated based on the volume changes per mmHg during the distension, while the gastric sensitivity is presented as the rate of change root mean square of the myoelectricity.

### Ussing chamber experiment

The duodenal epithelial barrier integrity was examined by Ussing chamber system (Physiologic Instruments, USA). After the muscular layer and serosa were removed, the duodenal mucosal layer was mounted in the Ussing chamber as described previously [2]. The chambers were filled with 5 ml Krebs buffer that was maintained at 37 °C and perfused with O_2_/CO_2_ (95/5%) continuously. After a 30-min equilibration period, a constant electric pulse of 1 mV every 60 s with duration of 0.2 s started and the voltage deflection was recorded every 15 min over 1-h duration. The average voltage deflection of the four time points was calculated and Transepithelial electrical resistance (TEER) is presented as Ω/cm^2^.

### Western blot

The duodenum was homogenized by using a bead mill (Biospec, USA) in RIPA buffer containing 1 mM PMSF (Solarbio, China). The BCA assay was used to measure the protein concentration. Equal amount of total proteins (80 μg/lane) was loaded on SDS-PAGE gel and transferred to PVDF membrane after separation. The membrane was then incubated with the following primary antibodies: anti-occludin (1:400, Santa Cruz Biotechnology), anti-claudin-1(1:2000, Abcam), and anti-β-actin (1:1500, ZSGB-BIO) at 4 °C overnight. After three 10-min washing with PBS (supplemented with 0.1% Tween 20), the membrane was incubated with appropriate secondary antibodies: HRP-conjugated goat anti-mouse, rabbit anti-goat or goat anti-rabbit (both 1:5000, ZSGB-BIO) antibody. The protein bands on the membrane were visualized following incubation with enhanced chemiluminescence substrate and the protein levels were quantified with Image J software.

### Immunofluorescence

Duodenum was embedded in OCT and frozen sections at 6-μm thickness were processed with freezing microtome (ThermoFisher, USA). Sections were incubated with anti-occludin (1:50, Santa Cruz Biotechnology) or anti-claudin-1 (1:40, Abcam) at 4 °C overnight. Following three 10-min washing, the sections were then incubated with rabbit anti-goat or goat anti-rabbit (1:100, ZSGB-BIO) secondary antibody at room temperature for 45 min. The slides were mounted with mounting medium with DAPI (ZSGB-BIO) and signal was examined under fluorescence microscope (OLYMPUS DP71, Japanese).

### Enzyme-linked immunesorbent assay (ELISA)

Samples were homogenized and the supernatants were collected. Total protein concentration was measured by BCA assay. The concentrations of tumor necrosis factor alpha (TNF-α) and interferon gamma (IFN-γ) were detected with Rat TNF-α and IFN-γ ELISA kits (Cusabio, China) respectively according to the manufacturer’s instructions. Briefly, TNF-α or IFN-γ antibody-coated plates were incubation with the above collected supernatants, followed by addition of biotin-conjugated antibody and avidin-conjugated HRP prior to incubation with TMB substrate. The signal was detected by a microplate reader (BioTek Instruments, USA) at 450 nm, and the concentration of TNF-α and IFN-γ is presented as pg/mg protein.

#### Quantitative real-time polymerase chain reaction (qPCR)

The RNA was extracted from the duodenum using the TRIZOL reagent (Thermo Fisher Scientific, USA) and 5 μg of total RNA were reverse transcribed to cDNA according to the manufacturer’s instructions. Equal amount of cDNA was used to examine the expression of transient receptor potential vanilloid type 1 (TRPV1) (β-actin as an internal control) by using the SYBR Master Mix (Promega, USA) and the CFX 96 Real-time PCR System (BIO-RAD, USA) under the conditions of initial activation at 95 °C for 2 min, 40 cycles of denaturation at 95 °C for 15 s, annealing/extension at 60 °C for 1 min. The primer sequences are: β-actin (predicted size: 150 bp): sense 5′-AGTTGCGTTACACCCTTTC-3′, antisense 5′-CACCTTCACCGTTCCAGT-3′; TRPV1 (predicted size: 262 bp): sense 5′-GACATGCCACCCAGCAGG-3′, antisense 5′-TCAATTCCCACACACCTCCC-3′.

### Statistical analysis

All data were expressed as mean ± SEM and one way analysis of variance (ANOVA) was used to test the statistical significance among the groups. Statistical significance was defined by *P* < 0.05. SPSS 16.0 was used for all statistical analyses.

## Results

### SNS ameliorates visceral hypersensitivity (VH) and enhances gastric compliance in FD rats

**Fig. 1:**
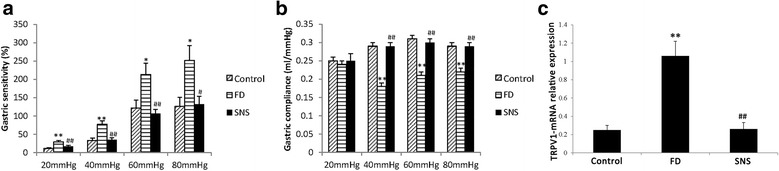
Visceral sensitivity (**a**), gastric compliance (**b**) and mRNA expression of TRPV1 (**c**). The model group had elevated visceral sensitivity and decreased gastric compliance compared with the control group. SNS treatment was effective to recover the visceral sensitivity and gastric compliance. The mRNA level of TRPV1 was notably increased in the duodenum of the model group compared with the control group, and the SNS treatment decreased the expression of TRPV1. All data were represented as mean ± SEM (6 rats in each group).^*^ *P* < 0.05 compared with the control group; ^**^ *P* < 0.01 compared with the control group; ^#^ *P* < 0.05 compared with the model group. ^##^ *P* < 0.01 compared with the model group

VH is an underlying cause of FD symptoms, whereas decreased gastric compliance is known to be a consequence of FD. Herein, we found that FD rats elicited VH under all distention pressures of 20 mmHg (29.22 ± 3.59 vs. 11.91 ± 1.68, *P* < 0.01), 40 mmHg (77.31 ± 9.30 vs. 33.44 ± 6.64, *P* < 0.01), 60 mmHg (213.39 ± 30.65 vs. 122.08 ± 21.28, *P* < 0.05) and 80 mmHg (251.32 ± 40.72 vs. 126.40 ± 24.61, *P* < 0.05) compared with the control group (Fig. 1a). Importantly, SNS treatment significantly decreased visceral sensitivity in FD rats at 20 mmHg (16.81 ± 3.05 vs. 29.22 ± 3.59, *P* < 0.01), 40 mmHg (35.00 ± 5.80 vs. 77.31 ± 9.30, *P* < 0.01), 60 mmHg (106.54 ± 11.51 vs. 213.39 ± 30.65, *P* < 0.01), and 80 mmHg (132.50 ± 20.93 vs. 251.32 ± 40.72, *P* < 0.05) (Fig. 1a). On the other hand, compared with the control animals FD rats showed a significant decrease in gastric compliance at 40 mmHg (0.18 ± 0.01 vs. 0.29 ± 0.01, *P* < 0.01), 60 mmHg (0.21 ± 0.01 vs. 0.31 ± 0.01, *P* < 0.01) and 80 mmHg (0.22 ± 0.01 vs. 0.29 ± 0.01, *P* < 0.01), but not at 20 mmHg (0.21 ± 0.01 vs. 0.25 ± 0.01, *P*>0.05) (Fig. 1b). Treating FD rats with SNS significantly improved gastric compliance under the pressures of 40 mmHg (0.29 ± 0.01 vs. 0.18 ± 0.01, *P* < 0.01), 60 mmHg (0.30 ± 0.01 vs. 0.21 ± 0.01, *P* < 0.01) and 80 mmHg (0.29 ± 0.01 vs. 0.22 ± 0.01, *P* < 0.01) (Fig. 1b). These findings thus suggest that SNS improves FD symptoms, which is probably through attenuation of gastric hypersensitivity. To further confirm that SNS improves VH, we analyzed the expression of TRPV1, which is a key factor that meditates VH. The qPCR result showed that the mRNA level of TRPV1 was significantly increased in the duodenum of FD rats as compared to the control group (1.06 ± 0.16 vs. 0.25 ± 0.05, *P* < 0.01) (Fig. 1c). Importantly, SNS treatment significantly reduced TRPV1 expression in FD rats (0.26 ± 0.07 vs. 1.06 ± 0.16, *P* < 0.01).

### SNS improves TEER in FD rats

**Fig. 2:**
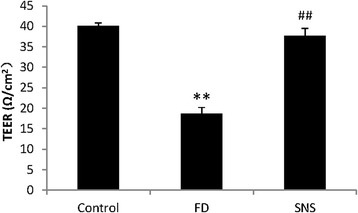
Duodenal TEER. The model group had a prominently decreased TEER compared with the control group, and SNS treatment remarkably enhanced the TEER level of FD rats. All data were represented as mean ± SEM (6 rats in each group). ^**^ *P* < 0.01 compared with the control group; ^##^ *P* < 0.01 compared with the model group

Impaired duodenal function is known to cause gastric hypersensitivity and FD symptoms [16–18]. It has recently been shown that duodenal barrier function is compromised in FD. We asked whether TEER is altered in our FD rats, and assessed the potential effects of SNS on TEER regulation. As shown in Fig. 2, TEER values as detected by Ussing chamber was significantly decreased in the duodenum of FD rats as compared with the control group (18.71 ± 1.51 vs. 40.06 ± 0.77, *P* < 0.01). SNS treatment remarkably enhanced the TEER level of FD rats (37.70 ± 1.84 vs. 18.71 ± 1.51, *P* < 0.01), indicating that SNS can repair duodenal epithelial barrier function.

### SNS increases the expression of TJ proteins in FD rats

**Fig. 3:**
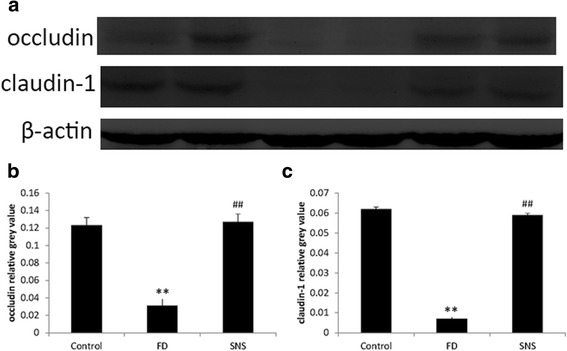
Representative Western blot bands of TJ proteins (**a**).The expression level was normalized to the housekeeping protein β-actin (**b**, **c**). Compared with the control group, the expression of occludin and claudin-1 was significant lower in the model group and SNS treatment could restore these two TJ proteins. All data were represented as mean ± SEM (6 rats in each group). ^**^ *P* < 0.01 compared with the control group; ^##^ *P* < 0.01 compared with the model group

**Fig. 4:**
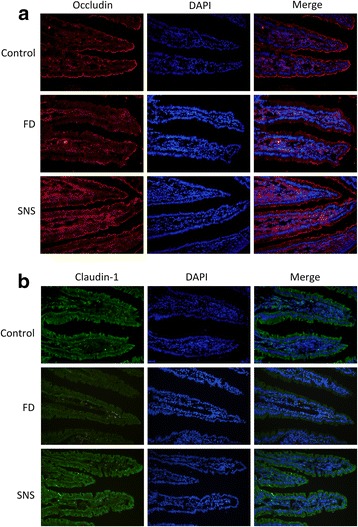
Representative photomicrographs of immunofluorescence of occluding (**a**) and claudin-1 (**b**). These pictures showed a decline in the expression of both occluding and claudin-1 in the model group, which was restored in response to SNS treatment

TJ proteins create the first line of paracellular barrier, protecting the mucosa from the external environment. We next determined whether SNS restores TEER by regulating of the expression of TJ proteins. Occludin and claudin-1 were examined in the present study because of their essential roles in maintaining a tight barrier [19]. Western blot analysis showed that the expression levels of occludin and claudin-1 were significant lower in the FD rats as compared with the control group (occludin: 0.031 ± 0.008vs. 0.123 ± 0.009, *P* < 0.01; claudin-1: 0.007 ± 0.001 vs. 0.062 ± 0.001, *P* < 0.01) (Fig. 3). Moreover, immunofluorescence staining showed a decrease in the expression of both occludin (Fig. 4a) and claudin-1 (Fig. 4b) in the FD group, which was restored in response to SNS treatment. These findings implicate that SNS enhances epithelial barrier function at least in part through the up-regulation of TJ proteins.

### SNS attenuates the expression of pro-inflammatory cytokines in FD rats

Impaired duodenal barrier function exposes the body to luminal environment where commensal bacteria inhabit, which can cause inflammatory response in the mucosa. In fact, several studies have demonstrated the presence of low-grade inflammation in the duodenum of FD patients [20, 21]. In this study, we assessed whether application of SNS in FD rats modulates the inflammatory state by examining the expression of typical pro-inflammatory cytokines, TNF-α and IFN-γ.

**Fig. 5:**
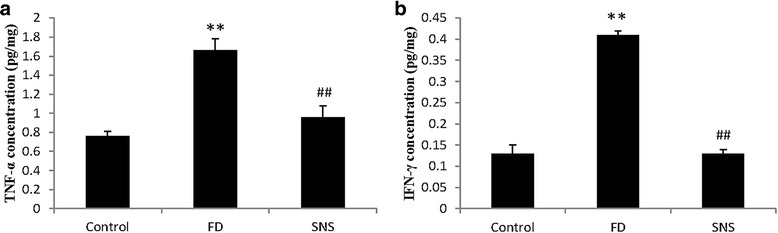
Concentrations of TNF-α (**a**) and IFN-γ (**b**) in the duodenum. TNF-α and IFN-γ levels were remarkably higher in the duodenal mucosa of model group than the control group. The SNS group showed a lower level of expression of TNF-α and IFN-γ.. All data were expressed as mean ± SEM (6 rats in each group). ^**^ *P* < 0.01 compared with the control group; ^##^ *P* < 0.01 compared with the model group

TNF-α and IFN-γ levels were significantly higher in the duodenum of FD rats than in the control (TNF-α: 1.66 ± 0.12 vs. 0.76 ± 0.05, *P* < 0.001; IFN-γ: 0.41 ± 0.01 vs.0.13 ± 0.02, *P* < 0.01). Importantly, upon SNS treatment, FD rats showed a much lower level of TNF-α and IFN-γ (TNF-α: 0.96 ± 0.12 vs. 1.66 ± 0.12, *P* < 0.01; IFN-γ: 0.13 ± 0.01 vs. 0.41 ± 0.01, *P* < 0.01), indicating that SNS could ameliorate micro-inflammation in the duodenum of FD rats (Fig. 5).

## Discussion

Our study demonstrated that SNS treatment attenuates FD-associated VH and improves gastric compliance. The underlying therapeutic mechanisms are that SNS improves duodenal barrier integrity by increasing occludin and claudin-1 expression and suppresses micro-inflammation as suggested by decreased expression of TNF-α and IFN-γ in the duodenum.

The intestinal barrier is critical in preventing the translocation of noxious substances and dietary antigens from the gut lumen to the submucosa. Although a large body of evidence has clearly demonstrated intestinal barrier dysfunction is closely related to intestinal diseases including ulcerative colitis [22] and irritable bowel syndrome (IBS) [23], a pathologic relationship between duodenal barrier and FD has only been recognized recently [2, 24]. Our finding of decreased TEER in FD rats is in accordance with previous reports. The TJ proteins are essential components of the intestinal barrier, and consist of transmembrane proteins, such as occludin, claudin-1, and scaffolding proteins, such as zonula occludens (ZO) [19]. Claudins are the key factors of the paracellular seal, by interacting with ZO-1 or self-organization [25]. Occludin was the first identified TJ transmembrane protein [26]. Knockdown of cccludin results in an increase of permeability and the down-regulation of occludin was implicated in intestinal diseases [27]. The mechanisms by which tail clamping induces barrier dysfunction remains unclear; however, clinical trials and animal experiments both showed that acute stress has a significant influence on the intestinal inflammatory response and the expression of TJ proteins [28, 29]. Of note, we observed that the expression of occludin and claudin-1 was significantly decreased in FD rats. It is likely that the decrease in occludin and claudin-1 expression in the duodenum of FD rats is induced by tail clamping-associated stress through yet unknown mechanisms, such as changes in hormone secretion and/or signaling transduction. The most important finding of the present study is that oral administration of SNS enhances the expression of TJ protein occludin and claudin-1 in the duodenum, which highlights the potential molecular basis for the therapeutic role of SNS in FD treatment. One of the potential mechanisms is that SNS treatment inhibits proteolysis of occludin by suppressing the transcription of matrix metalloproteinase (MMP) [30, 31].

The impairment of duodenal integrity leads to abnormal penetration of toxic substances, including bacterial metabolites and dietary antigens, which result in activation of the autoimmune system and increased infiltration of inflammatory cells. Many studies have demonstrated the abnormal infiltration of mast cells, eosinophil, and intraepithelial lymphocytes in FD patients [2, 21, 29, 32]. Immune cell infiltration-mediated inflammatory response might account for our current observation of increased amount of TNF-α and IFN-γ. This finding in murine model is consistent with a previous report of human FD patients. Given the stimulatory effect of SNS on the expression of occludin and claudin-1, it is likely that decreased expression of inflammatory cytokines in SNS-administered FD rats results from barrier restoration. However, we can not rule out the possibility that SNS exerts direct effects on immune cell behavior attenuating inflammatory response. It has previously been reported that SNS decreases TNF-α expression in trinitrobenzene sulfonic acid-induced pancreatitis [33], which does not seem to involve barrier restoration. Future study by measuring temporal changes of TEER vs. inflammatory cytokine expression during SNS treatment should provide a better clue. It is also important to test if SNS application in human FD patients enhances duodenal barrier function, and to understand the precise mechanisms of SNS-mediated up-regulation of occludin and claudin-1 expression by using animal or cell culture models.

Notably, another key finding of our study is that SNS attenuates FD-associated VH. TRPV1 is a primary causal factor for VH, and many studies have demonstrated the positive co-relationship between TRPV1 expression and VH [34]. It remains unclear how SNS treatment attenuates TRPV1 expression and improves VH in FD rats. It has previously been shown that the duodenum with impaired TJ shows hypersensitivity to acid and lipid [16, 17], which can provoke visceral afferents [18]. Moreover, a leaky TJ leads to the invasion of luminal microflora and their metabolites that stimulate immune response as we observed in the current study – the increased expression of TNF-α and IFN-γ. In fact, it has been demonstrated that the expression of TRPV1 is up-regulated in inflammation [35], and TRPV1 can further augment inflammation by activating STAT3 signaling [36]. Therefore, we speculate that restored TJ function in response to SNS has an important role in ameliorating VH and FD symptoms.

## Conclusions

Our study demonstrated that increased expression of TRPV1, decreased expression of TJ proteins, and the subsequent low-grade inflammation are associated with FD. Importantly, we showed for the first time that Chinese herbal medicine SNS ameliorates the severity of FD possibly through restoration of normal duodenal barrier function by up-regulating occludin and claudin-1 expression and attenuation of local low-grade inflammation as evidenced by decreased secretion of TNF-α and INF-γ. Our findings thus shed novel light on the molecular basis that underlies SNS-mediated therapeutic effects in FD treatment.

## Additional file

Additional file 1: Dataset.ᅟ(XLSX 13 kb)

## Abbreviations

ELISA: Enzyme-linked immunesorbent assay
FD: Functional dyspepsia;
IBS: Irritable bowel syndrome
IFN-γ: Interferon gamma
MMP: Matrix metalloproteinase
SNS: Sini-San
TCM: Traditional Chinese medicine
TEER: Transepithelial electrical resistance
TJ: Tight junction
TNF-α: Tumor necrosis factor alpha
TRPV1: Transient receptor potential vanilloid type 1
